# Thermogenic adipocytes: lineage, function and therapeutic potential

**DOI:** 10.1042/BCJ20200298

**Published:** 2020-06-12

**Authors:** Alice E. Pollard, David Carling

**Affiliations:** 1 MRC London Institute of Medical Sciences, Imperial College London, Hammersmith Hospital, London W12 0NN, U.K.; 2 Structure Biophysics and Fragments, Discovery Sciences, Biopharmaceuticals R&D, AstraZeneca, Cambridge, U.K.; 3 Institute of Clinical Sciences, Imperial College London, Hammersmith Hospital, London W12 0NN, U.K.

**Keywords:** adipocytes, brown adipose tissue, thermogenesis, white adipose tissue

## Abstract

Metabolic inflexibility, defined as the inability to respond or adapt to metabolic demand, is now recognised as a driving factor behind many pathologies associated with obesity and the metabolic syndrome. Adipose tissue plays a pivotal role in the ability of an organism to sense, adapt to and counteract environmental changes. It provides a buffer in times of nutrient excess, a fuel reserve during starvation and the ability to resist cold-stress through non-shivering thermogenesis. Recent advances in single-cell RNA sequencing combined with lineage tracing, transcriptomic and proteomic analyses have identified novel adipocyte progenitors that give rise to specialised adipocytes with diverse functions, some of which have the potential to be exploited therapeutically. This review will highlight the common and distinct functions of well-known adipocyte populations with respect to their lineage and plasticity, as well as introducing the most recent members of the adipocyte family and their roles in whole organism energy homeostasis. Finally, this article will outline some of the more preliminary findings from large data sets generated by single-cell transcriptomics of mouse and human adipose tissue and their implications for the field, both for discovery and for therapy.

## Introduction

The dramatic rise in the incidence of metabolic disease has promoted a major increase in adipose tissue research over the last decade. The loss of metabolic flexibility in lipid-storing tissues is a driving force behind complications in obesity, type II diabetes and cardiovascular disease that together contribute to the metabolic syndrome, one of the leading causes of death worldwide [1–3]. Whilst dietary intervention and promotion of an active lifestyle remain arguably the most effective preventative measures to combat these diseases, access to high-calorie, nutrient-poor foods and an increasingly sedentary societal infrastructure are increasing the demand for alternative therapeutic approaches. Previous efforts aimed at developing drugs to treat aspects of the metabolic syndrome focused on reducing caloric intake, both through appetite suppression and restricting absorption of lipids and carbohydrates in the gut. More recent efforts have begun to approach the problem from the other side of the energy-balance scale, through increasing metabolic rate.

At the forefront of this research is the promotion of adipose tissue-mediated thermogenesis, both in classical brown adipose tissue (BAT) and through the formation of brown-like adipocytes in white adipose tissue (WAT) depots. Whilst the ability to induce brown adipose characteristics in white adipocytes is now widely accepted, little is understood about the origin of these cells, and even less so the importance of their origin to their function. Advances in RNA sequencing technology has provided evidence for the existence of multiple adipocyte subtypes, defined not merely by morphology, but by their cellular origin and ability to adapt to metabolic stress. Lineage tracing has allowed us to begin to dissect the heterogeneity of adipose depots, with some proving to be far more complex than previously anticipated. Combining these powerful techniques, together with complimentary -omics, substrate labelling and advanced imaging strategies, has brought adipocyte biology to the forefront of metabolic research.

These exciting findings, though in their infancy, raise an important question deserving of investigation; does lineage contribute to the function and plasticity of an adipocyte? This article will discuss the structural and functional characteristics of both classical and novel adipocyte subtypes, their developmental origin and how these may come together to regulate whole organism energy homeostasis. The therapeutic potential of these different cell types is yet to be determined, but with an increasing interest in the adipocyte field from both basic and translational perspectives, the next decade of adipocyte research is well placed to deliver exciting new findings.

## Adipocytes: masters of energy homeostasis

Until recently, white adipocytes were thought of as metabolically inert lipid storage cells, and were often referred to simply as fat cells. Now it is recognised that adipocytes encompass a highly heterogeneous, plastic and metabolically active diverse array of cell types. Adipocytes are found both in discrete depots and interspersed in other organs. Different types of adipocytes can be distinguished according to their appearance (colour) together with their gross cellular characteristics (e.g. number of mitochondria, size and number of lipid droplets). In many cases, this classification is used to assign different functional attributes, such as thermogenesis. We now appreciate that there are different adipocyte precursor cells with distinct lineages and that these lead to adipocyte cell types with discrete and varying functions. This newly gained understanding raises important questions regarding the relationship between adipocyte cell lineage and function, and opens up avenues for therapeutically targeting specific populations of adipocytes as a way to treat metabolic disorders, such as obesity and type 2 diabetes.

## White adipose tissue: beyond lipid storage

**Figure 1.**
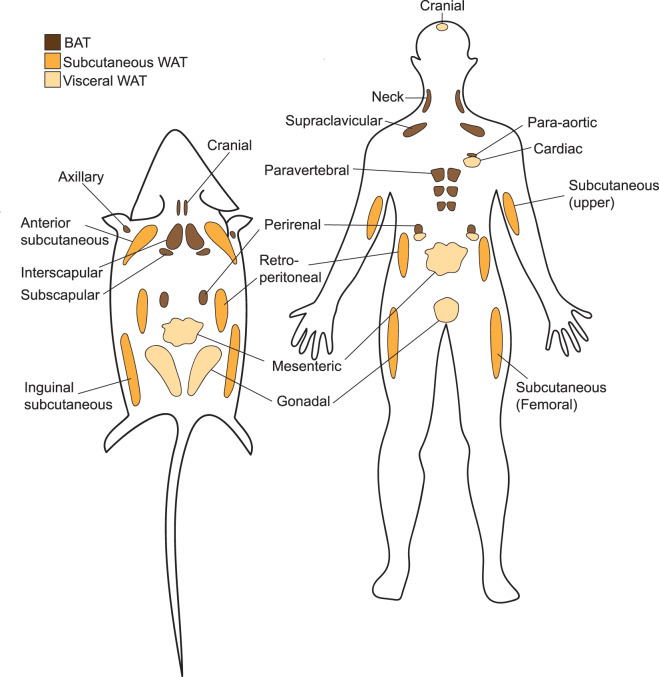
Anatomical location of adipose tissue depots. The locations of different depots of brown (BAT), subcutaneous and visceral white adipose tissue (WAT) in mice and humans is shown.

Classical white adipocytes are unilocular, containing one large lipid droplet serving as a store for triglycerides, with the capacity to expand and contract in response to energy demand [4,5]. WAT depots are distributed throughout the body, with nomenclature differing between species and confusingly, sometimes between individual studies. In rodents, visceral (trunk) depots include the perigonadal (pgWAT), retroperitoneal (rWAT) and mesenteric (mWAT). Subcutaneous adipose depots (scWAT) are divided into the anterior subcutaneous (asWATs), namely the interscapular and axillary WATs, and the inguinal WAT (iWAT) located dorsally, attached to the hindlimb and pelvis [4,6]. These rodent depots and their counterparts in humans are outlined in Figure 1. White adipocytes are also found dispersed in the periphery, with smaller discreet depots including intramuscular [7–9] and dermal [10–12] adipocytes now emerging as important local regulators of tissue function. Depot-specific responses to metabolic alterations caused by diet, age, hormone signalling and disease have been reported, with an increase in visceral adipose mass associated with metabolic disease [13–18]. In contrast, an increase in scWAT correlates with a reduced disease risk [14]. Insights such as these suggest functional differences exist between populations of white adipocytes, influenced by their ability to respond to external stimuli. Thus, an understanding of the origin of individual adipocyte populations within each depot and their respective function will increase our understanding of their contribution to health and disease.

A common function of WAT, regardless of anatomical location, is to store and release triglycerides in response to whole body energy demand [13,18–22]. Retrieval of stored lipids is facilitated by a layer of specialised lipid-associated proteins from the perilipin family [23], enabling recruitment of hormone sensitive lipase (HSL), adipose triglyceride lipase (ATGL) and monoacylglycerol lipase (MAGL) to catalyse lipid breakdown [24] ^1^
^1^For ease of reading, throughout this review, protein and gene names will be depicted in uppercase regardless of species.. The existence of perilipin 1 in association with lipid droplets in white adipocytes serves to restrict lipolysis under basal conditions, as well as to create a barrier between otherwise toxic lipid species with surrounding cells [23]. Loss of perilipin 1 leads to increased basal lipolysis, inflammatory cell recruitment denoted by formation of crown-like structures, and ultimately cell death. Several inflammatory stimuli, including tumour necrosis factor α (TNFα) and interleukin 6 (IL6), contribute to increased immune cell infiltration and loss of perilipin 1 in obese individuals, resulting in impaired lipolysis and triglyceride storage in WAT [25,26]. Loss of adipocyte plasticity under these conditions drives peripheral lipid accumulation and contributes to the development of systemic insulin resistance. Preservation of lipid droplet integrity and reduction in pro-inflammatory cytokine release in WAT is therefore critical for the maintenance of normal white adipocyte function, extensively reviewed in [23].

In addition to lipid storage, white adipocytes contribute to whole organism energy homeostasis through the production and secretion of endocrine and paracrine factors, and are themselves sensitive to extracellular signalling [19,27–29]. Insulin, a central regulator of carbon deposition and metabolism, drives glucose uptake in adipocytes and stimulates fatty acid synthesis through increased expression of fatty acid synthase (FAS) [16,17,27]. In cell culture, and *in vivo*, insulin drives the adipogenic gene programme in adipocyte precursors, through the stimulation of sterol regulatory element-binding protein (SREBP)-1c [30] and up-regulation of the master regulator of adipogenesis peroxisome proliferator-activated receptor gamma (PPARγ) via mammalian target of rapamycin complex 1 (mTORC1) activation [31–33]. Insulin/IGF1 (insulin-like growth factor 1) knockout (KO) mice display significant reduction in adipose tissue mass with defective basal thermogenic capacity [33]. Expression of the insulin receptor (INSR) and intact IGF-1 signalling are therefore critical for the commitment of adipocyte stem cells to adipogenesis and for the maintenance and function of mature adipose tissue [33]. Variations in insulin sensitivity within adipocyte precursor populations is one of several proposed determinants of lineage and cell fate. Deletion of tumour suppressor phosphatase and tensin homologue (*PTEN*), the negative regulator of insulin/phosphatidylinositol 3-kinase (PI3K) growth stimulating pathways, in a subset of adipocyte precursors, leads to aberrant signalling independent of ligand binding [34,35]. The resulting increase in glucose transport provides a metabolic advantage in targeted cells, driving proliferation, hypertrophy and redistribution of adipose mass. Insulin is just one of several factors implicated in the organisation and selective proliferation of adipocyte precursors. Alterations in adipocyte-derived cytokines (adipokines), including leptin, adiponectin and TNFα are common under obesogenic conditions [16,36–45], causing changes in food intake, impaired satiety response and chronic inflammation [1,46–52]. Infiltration of pro-inflammatory immune cell populations, forming distinctive crown-like structures [53], is a hallmark of obesity and is often associated with onset of insulin resistance, diabetes and loss of metabolic flexibility. The role of WAT in metabolic flexibility is evident, and is reviewed extensively for its hormonal and lipid-storing functions in health and disease. Many studies targeting adipose tissue derived stem cells have benefited from its abundance in mice and humans, and are now beginning to dissect its extraordinary heterogeneity. The following sections will discuss additional adipocyte populations and their contributions to whole organism energy homeostasis.

## Brown adipose tissue: UCP-1 mediated thermogenesis and beyond

BAT is highly innervated, highly vascularised and metabolically active [54–58]. Discrete depots are located in the interscapular (iBAT), sub-scapular (sBAT) and cervical (cBAT) regions, and smaller depots have been reported in association with the kidneys and aorta [6,59–63] (shown in Figure 1). Brown-like or ‘beige/brite’ adipocytes, hereafter referred to as beige adipocytes, exhibit many features characteristic of brown adipocytes. Importantly, for the purpose of their classification, beige adipocytes are distinguished by their anatomical location, interspersed in WAT depots. The number of beige adipocytes increases markedly in response to cold-exposure, a phenomenon known as ‘browning’. Most studies indicate that beige adipocytes derive from a white adipocyte precursor stemming mainly from the scWAT depot.

**Figure 2.**
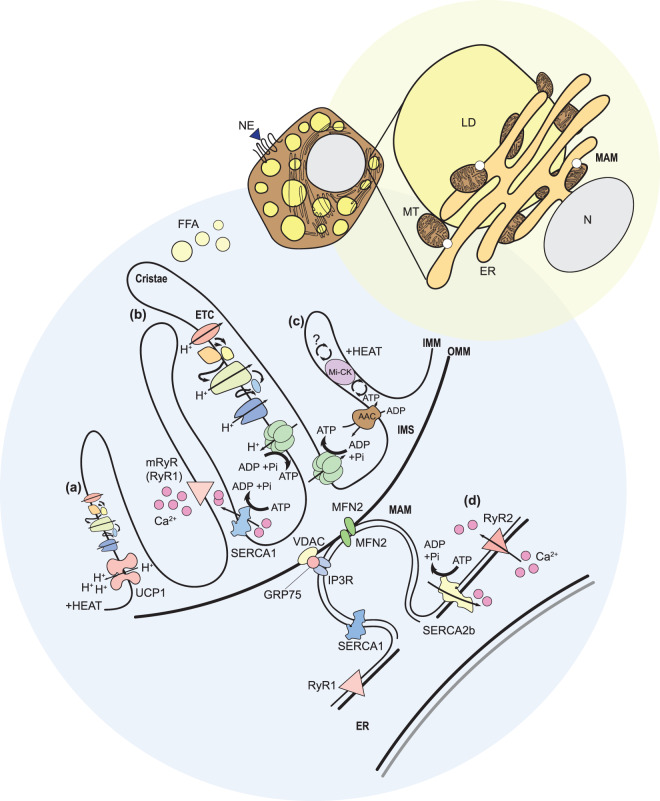
Thermogenic mechanisms in BAT and WAT. Brown adipocyte architecture contributes to thermogenic phenotypes through vascularisation, innervation, multilocular lipid droplets (LD) and high mitochondrial (MT) density. Assembly of mitochondria-organelle networks facilitate substrate utilisation, including the formation of mitochondria-associated ER membrane tethers (MAM) by protein bridges [64,90–96]. (**a**) Canonical UCP1-mediated thermogenesis via uncoupling of the mitochondrial electron transport chain (ETC), resulting in H^+^ gradient disturbance and proton leak, dissipating energy as heat. UCP1 activity is stimulated by free fatty acid (FFA) and inhibited by purine nucleotides [65,67,69,97–105]. (**b**) SERCA/RyR mediated Ca^2+^ futile cycling in mitochondria. SERCA1 is found in the inner mitochondrial membranes (IMM) of BAT [64,106]. Ca^2+^ enters the mitochondria via mitochondrial Ca^2+^ transporters and is pumped into the inner mitochondrial space (IMS) by SERCA1, with concomitant ATP hydrolysis. Ca^2+^ returns to the matrix via RyR. These cycles are abundant in the ER of brown adipose, heater organs (fish) and skeletal muscle [64,106–113]. A leaky mitochondrial RyR drives increased ATP hydrolysis uncoupled from net Ca^2+^ transport generating heat. This may also be subject to an unknown uncoupling agent [114]. (**c**) Non-canonical thermogenesis through creatine futile cycling. ATP generated by the ETC is shuttled into the IMS by the ATP transporter AAC in return for ADP. ATP is then hydrolysed by mitochondrial creatine kinase (mi-CK) to drive creatine phosphorylation to PCr. The reversal, driven by an as yet unidentified enzyme completes the futile cycle [115–118]. (**d**) Non-canonical thermogenesis by SERCA2b-driven Ca^2+^ futile cycling on the ER [114,119–121]. See text for more details.

Classical brown adipocytes are hexagonal cells, containing many small lipid droplets (multilocular) and are rich in mitochondria. Brown adipocytes have an extensive endoplasmic reticulum (ER) network that forms contact points with the mitochondria, known as the mitochondria-associated ER membrane (MAM) [64,65]. The primary function of ‘classical’ BAT is widely accepted as non-shivering thermogenesis (NST). This function is enabled by sympathetic innervation, high mitochondrial number and mitochondrial specialisation [66], abundant lipid stores and by the expression of the respiratory chain uncoupling protein 1 (UCP1) [67–70] (see Figure 2). Heat production at the expense of ATP synthesis is in stark contrast with the primary function of white adipocytes, yet both play integral parts in global energy homeostasis, serving as both direct and indirect buffers of nutritional demand and excess.

Since the discovery of functional BAT in humans [71–73], extensive studies have reviewed its role in the context of health and disease, and as a potential target for the treatment for metabolic disease through the activation of UCP1. Uncoupling of the mitochondrial respiratory chain by UCP1 serves to increase heat production during cold exposure, particularly in hairless neonates, and provides resistance to obesity resulting from overfeeding. Both functions require exquisite sensitivity to extracellular signalling, mediated by dense vascularisation and innervation [74–78]. Perhaps the most well studied of these cascades is the initiation of thermogenesis by adrenergic receptor (AR) signalling. Responses to adrenaline and nor-adrenaline are co-ordinated by ARs, which are members of the G protein-coupled receptor super-family. Brown adipocytes express high levels of β3-ARs which couple to Gs proteins, leading to activation of adenylate cyclase and an increase in intracellular cyclic AMP levels. Cyclic AMP induces gene expression mediated by the transcription factor, cyclic AMP-response element binding protein (CREB) [79]. Brown adipocytes also express α2-ARs, which couple through Gi proteins inhibiting adenylate cyclase, counteracting β3-AR activation of thermogenesis. However, in rodents, β3-AR expression is much higher than α2-AR expression, and so adrenergic-signalling stimulates thermogenesis [80] Stimulation of AR-responsive gene expression is also brought about, at least in part, through p38-mitogen activated protein kinase (p38 MAPK) mediated phosphorylation of activating transcription factor 2 (ATF-2) [81–83], the histone demethylase jumonji domain-containing 1A (JMJD1A) [84,85] and peroxisome proliferator-activated receptor gamma co-activator alpha (PGC1α) [86,87]. PGC1α, once phosphorylated, is free to interact with PPARγ, facilitating the formation of a transcriptional complex with phosphorylated JMJD1A and SWItch/Sucrose Non-Fermentable (SWI/SNF) in adipocyte nuclei, bringing promotor, enhancer and coding regions into proximity, driving beige and brown-specific gene expression [85,88,89].

Extensive literature covers the role and regulation of UCP1-dependent thermogenesis in brown adipose tissue [65,67,69,97–100,122–127]. Based largely in small mammals (rodents), these studies have focused on UCP1 as the principle heat-generating pathway utilised by BAT in response to adrenergic signalling (Figure 2). Ablation of BAT by diphtheria toxin resulted in the onset of obesity and diabetes, coupled with hyperphagia, when mice were housed at ambient temperature. In contrast, global deletion of *UCP1* did not recapitulate this phenotype [128], demonstrating that functional BAT, but not UCP1 itself, is required for the prevention of metabolic disease. Supporting this, many studies have characterised UCP1-independent thermogenic mechanisms in both brown and beige adipocytes [67,114–116,119,129–135].

## UCP1-Independent thermogenic mechanisms in adipose tissue

The existence of UCP1-independent NST mechanisms is well established, particularly in skeletal muscle [136–138] and, to a lesser extent, in BAT [107–109,139], and provides insight into the origins of endothermy in mammals. Importantly, similar mechanisms have been identified in specialised beige and white adipocytes [115–117], and promotion of these mechanisms leads to improved metabolic health, opening new avenues for the treatment of metabolic disease. Evolutionary evidence suggests that these mechanisms pre-date the emergence of UCP1, a defining feature of BAT, with many species entirely reliant on their existence to facilitate high core body temperatures [136,140]. The hydrolysis of ATP to ADP provides the energy to drive virtually all of the biological processes required to maintain life. However, in addition to providing energy for biological work, the energy from ATP hydrolysis can be converted to heat [108,139,140]. Increased ATP hydrolysis can occur in a futile cycle leading to increased heat production. This mechanism, best described in skeletal muscle, utilises a calcium (Ca^2+^) futile cycling mechanism involving the ryanodine receptor (RyR) Ca^2+^ channel and the sarco/endoplasmic reticulum Ca^2+^ ATPase (SERCA) can be used for thermogenesis during cold-stress [136,141]. The sarcoplasmic reticulum (SR), a membranous network in muscle analogous to the endoplasmic reticulum, is responsible for the initiation of muscle contraction and can store Ca^2+^ in millimolar concentrations. Muscle contraction is initiated by the release of Ca^2+^into the cytosol by the RyR and relaxation occurs when Ca^2+^ is actively pumped back into the SR by SERCA. Although this Ca^2+^ cycling is coupled, a small amount of heat (∼10–25 kcal/mol ATP) is generated with each exchange [106,109,142,143]. In response to cold-stimulus, the binding of sarcolipin (SLN) to SERCA uncouples ATP hydrolysis from Ca^2+^ transport, at the expense of muscle contraction, generating additional heat. In the absence of *UCP1* in BAT, SLN expression is up-regulated in skeletal muscle, enhancing survival rates during cold exposure [144–147]. This compensation is reciprocal, with *SLN*-KO mice displaying increased UCP1 expression and browning of WAT. Loss of both mechanisms rendered mice extremely cold sensitive during acute exposure, but able to survive if exposed gradually, at the expense of all lipid stores [147]. This implies that the two mechanisms are complementary, and to an extent compensatory, in rodents. Another factor influencing heat production is the rate at which Ca^2+^ returns to the cytoplasm, through RyRs. The fundamental role of RyRs in Ca^2+^ driven thermogenesis is highlighted in the context of malignant hyperthermia, in which missense mutations in skeletal muscle RyR1 result in abnormal Ca^2+^ release in response to ligands, predominantly volatile anaesthetic agents, leading to uncontrolled heat production in skeletal muscle [148]. Although a rare disease, malignant hyperthermia during anaesthesia could often prove fatal, but can now be treated effectively with the RyR inhibitor, dantrolene [149]

In some species, the reliance on muscle-based NST is far greater than in mammals. Ca^2+^ cycling through SERCA/RyR as a functional thermogenic pathway occurs in the ocular regions of several species of large oceanic, deep diving fish [139,140]. Termed a ‘heater organ’, this structure consists of modified muscle cells that lack proteins required for muscle contraction. Instead, the cells have an extensive SR network with membranous stacks located between mitochondria. This unusual arrangement generates a large surface area of the SR allowing for high level expression of SERCA, together with close proximity to ATP synthesis in the mitochondria. These adaptations enable high rates of Ca^2+^ cycling to drive thermogenesis. The development of this specialised system enables the fish to maintain eye and brain temperature significantly above the surrounding water temperature, ensuring optimal function in cold environments. In addition, some birds maintain core temperatures in excess of 38°C with some reaching ∼44°C in flight. Studies in hummingbirds have revealed that a futile Ca^2+^ cycling thermogenic mechanism involving SERCA operates in muscle to facilitate warming following their daily periods of torpor [150].

Though Ca^2+^ futile cycling is dominant in skeletal muscle, several other substrate fluxes are critical for the maintenance of ATP production [151]. Lipid futile cycling through the lipolysis and re-esterification of free fatty acid (FFA) in white adipose is well documented, in both the presence and absence of UCP1 [152–155]. Data from cultured brown adipocytes suggests these mechanisms are also present in BAT, with little reliance on UCP1 [151]. However, as the maximal thermogenic capacity of these cells in culture is likely diminished, it remains unclear as to whether these mechanisms are efficacious *in vivo* in response to thermogenic demand. Evidence for both glucose and FFA uptake in BAT *in vivo* is convincing [63] with administration of a β3-agonist increasing BAT and beige FFA accumulation. However, further work using these improved imaging techniques are required to determine the relative contribution to thermogenesis in the absence of BAT UCP1 [63].

The reliance on UCP1 in BAT for thermogenesis *in vivo* is likely due to the low expression of ATP synthase (complex V of the electron transport chain) [151,156]. Non-canonical, UCP1-independent thermogenic mechanisms capable of maintaining core body temperature require significant ATP synthesis to drive heat production, limiting the contribution of these pathways in BAT over long periods [97,147,157]. However, many species express functionally inactive UCP1, and several are devoid of *UCP1* in their genome [74]. These evolutionary divergences date back as far as the Cretaceous period, and correlate with an increase in body mass and a relative reduction in surface area. Small mammals, such as mice, use UCP1 to facilitate NST during cold periods, at the expense of ATP synthesis otherwise required to build biomass [6,158]. Thus, the study of thermoregulation in mammals has expanded to include mechanisms that whilst historically overshadowed by UCP1, are present and active in brown and beige adipose, outlined in Figure 2. The newly defined role of NST mechanisms in thermogenic adipocytes are perhaps reflective of a shared lineage between brown adipocytes and myocytes; both cell types arise from a lineage distinct from white adipocytes, marked by the myogenic regulatory factor MYF5 [6,34,159–161]. Given the limited efficacy and potential side effects of UCP1 activation in a clinical setting, the possibility of manipulating adipocyte differentiation combined with increasing UCP1-independent thermogenic mechanisms provides potential avenues for therapeutic intervention in key areas of metabolic diseases, including type 2 diabetes and obesity.

## The adipocyte lineage: heterogeneity and functionality

As is often the case when attempting to define different cell types, the distinction between adipocyte subtypes is imprecise. The similarities between beige and brown adipocytes raises the question of what is the contribution of their lineage to function. Progenitor pools in white adipose depots are known to give rise to both white and beige adipocytes [6,34,158,162,163], capable of performing both storage and thermogenic functions. Whilst rodents possess distinct brown and beige cell identities, human brown adipocytes exhibit gene expression profiles similar to beige adipocytes in rodents [58,129,164,165]. This suggests human brown adipose may more closely resemble rodent beige adipocytes, emphasising the importance of understanding lineage determination in the development of adipocyte cell type. An important factor to take into account when studying brown and beige thermogenic adipocytes, and particularly when making comparisons between rodents and humans, is the influence of external temperature. Laboratory mice are typically housed at temperatures within the range of 19–23°C, which is ∼10°C below their thermoneutral zone [166]. The thermoneutral zone for a mammal corresponds to the temperature at which the minimal amount of energy is required to maintain body temperature, and for mice this is ∼29°C [167]. This means that most experiments conducted on standard laboratory housed mice are done under sub-thermoneutral conditions. As a consequence, mice respond by increasing thermogenesis, including NST, primarily in BAT. For humans, defining the thermoneutral zone is complicated due to the use of clothing, but under many conditions, humans live within the thermoneutral zone, and so do not require high rates of thermogenesis. Although the effect of external temperature on NST is well appreciated, it is likely that some of the inferences made between human and mouse thermogenic adipocytes are confounded by the temperature at which the studies were performed.

Given the heterogeneity of adipose tissue, the number of discrete depots and the broad functionality of mature adipocytes, it is not surprising that their respective progenitors share the same complexity. Recent studies combining single-cell RNA sequencing and fluorescent imaging techniques have identified a number of new populations of adipose-resident stem cells and ‘pre-adipocytes’ in transition [168–170]. Whether these are each capable of full adipogenesis is an active area of research and is already yielding exciting results. Importantly, many of the populations identified in mice have been found in human adipose tissue, though their contribution to the mature tissue is currently unknown [168,170]. The following section combines what is known so far of the lineages of specialised adipocytes with respect to their functionality, as well as providing an overview of the techniques used to elucidate the hierarchies in adipocyte stem cell niches.

## Between brown and white: an adipocyte for all seasons

Although we have significant knowledge of the morphology and function of brown and white adipose tissue, our understanding of their developmental origins are less clear. Identification of beige adipocytes in scWAT depots in response to stimuli associated with proliferation of BAT only serves to reinforce our incomplete view of the situation. Genome-wide surveys of isolated brown adipocytes revealed transcriptional regulators capable of promoting a brown adipose phenotype in pre-adipocytes. Studies later revealed that PR-domain containing 16 (PRDM16) was capable of complete induction of the brown adipose thermogenic programme (e.g. *UCP1, PGC1Α, ADRB3 (β3-adrenergic receptor), DIO2 (deiodinase 2)*) [160,171–175] and mitochondrial gene expression in cultured mesenchymal stem cells*.* As a result, in both mice and humans, PRDM16 is regarded as a master regulator of brown adipose identity. PRDM16 is also a powerful repressor of muscle differentiation, an effect that is stabilised by euchromatic histone-lysine N-methyltransferase 1 (EHMT1) [160,172–179]. Indeed, deletion of *PRDM16* or *EHMT1* reverts cells to a myogenic state, with the formation of myosin heavy chain (MHC) positive myotubes together with expression of skeletal muscle genes and a loss of functional BAT *in vivo* [160,174,176]. Conversely, ectopic expression of PRDM16 in white adipose tissue drives beige adipocyte formation through interaction with CCAAT-enhancer binding protein β (C/EBPβ) and PPARγ [86,87,89,180]. The interest in PRDM16 as a regulator of adipocyte fate and function has expanded beyond beiging, as genetic overexpression of PRDM16 in white adipocyte depots led to the identification of novel cell types [114,172,174]. At present, it is not known whether these new cell types are expressed in wild type mice, or whether their expression requires specific genetic backgrounds. A summary of adipocyte cell types that have been identified is shown in Table 1.

**Table 1:** Summary of adipocyte populations, location(s), specialised/stimulating factors and key regulators of cellular fate

Adipocyte (specialised)	Known Lineage Markers	Specialisation	Key regulators	References
White (classical)	PDGFRα^+^; PDGFRβ^+/−^; MYF5^+/−^ (depot-specific); SCA1^+^; MYH11^+^; CD34^+^; CD29^+^; CD24^+^; CD31^−^; LIN^−^	Adipokine production, lipid storage, endocrine, insulation	PPARγ, C/EBPα/β/δ, RXR, CtBP1/2, PRDM16, ZFP423	[4,6,35,181–183]
Dermal (dWAT)	Camp; Ccl4, classic WAT (see above)	Hair cycling, skin wound healing, immune response	CAMP	[10–12,184–188]
Beige/brite	PDGFRα^+/−^; PDGFRβ^+/−^; SCA1^+^; MYH11^+^;CD34^+^; CD29^+^; CD24^+^; CD31^−^; LIN^−^	Thermogenesis (UCP1), glucose uptake, mitochondrial respiration, creatine futile cycling	PPARγ, PRDM16 EHMT1, PGC1α, C/EBPα/β/δ, ZFP516, ZFP423 EBF2, BMP7	[6,86,97,115,129–131,158, 174,181–183,189–195]
Alt. Beige (iWAT)	PRDM16^++^; UCP1^−/−^; PDGFRα^+^; SCA1^+^; MYH11^+^;CD34^+^; CD29^+^; CD24^+^; CD31^−^; LIN^−^	Thermogenesis (Ca^2+^ futile cycling SERCA2b/RyR2), glucose uptake	PRDM16, PPARγ, EHMT1, PGC1α, C/EBPα/β/δ, ZFP516, EBF2, BMP7	[114]
g-beige (iWAT)	PDGFRα^+^ SMA^+^; PAX3^+^; CD34^+^; CD29^+^ MYOD1^Lin+^	Glucose metabolism, glycolysis (ENO1), UCP1	GABPα	[133]
Pink (mammary)	AP2^+^; WAP^+^; ELF5; epithelial	Milk production	Pregnancy (unknown)	[102,196–199]
SMART (iWAT)	MYF5/6^+^; PAX7^+^	Thermogenesis (Ca^2+^ futile cycling SERCA1/RyR1/3), glucose metabolism, mitochondrial activity	AMPK activity	[134]
BAT	MYF5^+^; EN1^+^; Pax7^+^	Lipid storage, Thermogenesis (UCP1 and Ca^2+^ futile cycling Serca1), glucose metabolism	PRDM16/3, PPARγ, EHMT1, PGC1α, C/EBPα/β/δ, ZFP516, EBF2, BMP7, KLF11/15, TLE3	[6,55,58,87,131,174,176, 179,189,194,200–205]

The classical beige adipocyte, most notably induced by acute cold exposure, bears a striking resemblance to brown adipocytes, in both morphology and function. These beige adipocytes could occur either by transdifferentiation of mature white adipocytes, or through the proliferation of specific pre-adipocytes from stem cell niches [4,6,15,119,129–132,189,190,196–198,206,207]. Regardless of their origin, the contribution of these cells to adaptive thermogenesis and whole animal physiology has been documented thoroughly, at least in part due to their resemblance to human BAT [97,129,170,191,208,209]. Though new evidence now challenges the singular definition of a beige adipocyte [6,34,210–212], a classical cell signature (platelet-derived growth factor (PDGFR)α^+^; stem cells antigen 1 (SCA1)^+^; myosin heavy chain 11 (MYH11)^+^; CD34^+^; CD29^+^; CD24^+^; CD31^-^; LIN^-^) is attributable to both beige and white adipose progenitors [6,162,163]. These are distinct from the adipose endothelial signature (CD34^+^; CD31^+^) cells that are required for the formation of vascular endothelium in adipose tissue *in vivo* [213]. The adipogenic capacity of these vascular cells may yet prove to be of interest, as classical BAT may be in part derived from a CD31^+^ lineage [214].

As active BAT is low in humans beyond infancy, the potential to induce a brown-like adipocyte in WAT offered new therapeutic possibilities for the treatment of metabolic disease. The use of positron emission tomography (PET) with [^18^F] fluoro-2-deoxy-glucose (^18^FDG) in human subjects demonstrated an inverse correlation between BAT mass and body mass index, fasting plasma glucose and adiposity [58,215]. Moreover, BAT mass increased during acute cold stress, demonstrating recruitment of brown adipose progenitors in humans. Refined studies using ^18^FDG-PET later showed significant uptake in peripheral human BAT depots, including in the posterior subcutaneous region [216–218]. Though largely observational, these studies demonstrated that brown adipocytes are more widespread in humans than originally appreciated, being expressed in both classical BAT and WAT depots. In addition, the studies revealed that in humans brown adipocytes are induced in response to cold exposure, similar to that seen in rodents [97,101,130,170,219].

Despite the extensive work carried out *in vitro* using primary cells from human WAT, most of the literature surrounding beige adipose *in vivo* is based on murine models, a bias introduced due primarily to practical limitations than by design. Case studies in cancer cachexia [220–224], severe burn injury [219,225], thyroid carcinoma [226,227] and obesity have reported induction of BAT activation and recruitment of beige adipocytes, but there are no convincing examples of pharmacological induction in humans. Several molecules shown to promote beiging in mice have failed to elicit detectable induction in humans. Irisin, an exercise-induced myokine was shown to have beneficial effects on metabolic parameters associated with browning, including enhanced energy expenditure, lowered blood glucose, and a reduction in adiposity. Circulating irisin levels were correlated with induction of thermogenic genes associated with beiging, including a 5–500-fold induction of UCP1 mRNA [228]. Other studies have since disputed the potential of irisin as a therapeutic strategy, based partly on the finding that circulating irisin levels are increased in obese patients. These discrepancies, reviewed in depth by Crujeiras et al. [229], are as of yet unresolved and warrant further investigation, particularly due to the finding that irisin is also an adipokine and therefore any association with adiposity must be carefully dissociated from fat mass itself [228–232]. Another circulating factor, fibroblast growth factor 21 (FGF21), gained similar traction as a potential browning agent. In addition to its production in the liver, FGF21 has been shown to be secreted from activated brown adipocytes, eliciting a robust increase in UCP1 expression in human neck adipocytes, with lower induction in scWAT [233].

To understand the potential therapeutic relevance of browning of white adipose to human health, there needs to be a distinction between activation of thermogenesis and promotion of substrate utilisation for metabolic work, with heat production simply a by-product. When assessing the contribution of UCP1-mediated thermogenesis in beige adipose, both in mice and in humans, it is important to consider UCP1 protein expression and relative mitochondrial activity, in addition to *UCP1* gene expression [234]. The stimulation of *UCP1* gene expression by cold exposure in BAT is modest when compared with the ∼100-fold induction seen in WAT [97,131,189,206,235]. However, these differences have little bearing on the actual contribution of the different tissues to total thermogenic capacity [130,132,236]. Rather, they reflect the fact that the level of *UCP1* mRNA in WAT is very low under normal experimental conditions. This is because mice are routinely housed at ∼20°C thus the contribution of thermogenesis originating from WAT is low, and UCP1 expression is barely detectable. Following cold-exposure, the fold-induction of UCP1 expression is therefore large, even though the absolute level of UCP1 is low relative to BAT. Examining the contribution of beige cells to whole organism thermogenesis and metabolism is more relevant than focussing on UCP1 induction alone [234,237].

Despite its prominent role in BAT and beige thermogenesis, the loss of UCP1 whilst detrimental in acute cold exposure, is compensated for if gradual cold-acclimatisation is afforded [132,235,238], demonstrating the existence of other, UCP1-independent, thermogenic pathways. A quantitative proteomic study of isolated mitochondria from adipose tissue of cold-expose mice, identified an arginine/creatine metabolic pathway as a beige adipocyte signature [115] . Based on these initial findings, further studies revealed the existence of a creatine-driven futile cycling mechanism contributing to thermogenesis in beige adipocytes [116–118]. The translational significance of these findings is yet to be explored, although cultured human brown adipocytes show sensitivity to creatine-cycling inhibition, and a subset of white adipocytes in abdominal adipose tissue appear to use this mechanism preferentially for thermogenesis [115,116,239–242]. Using a different approach, another study showed that adipose-specific transgenic expression of PRDM16 in UCP1 KO mice led to the enrichment of genes associated with glycolysis, the tricarboxylic acid cycle cycle and strikingly, cardiac muscle contraction, notably the Ca^2+^ cycling components SERCA2b and RyR2 [114]. Increased expression of SERCA2b and RyR2 is suggestive of the futile Ca^2+^ cycling mechanism used for thermogenesis in skeletal muscle, described earlier in this article. It is noteworthy, however, that the cardiac isoforms (SERCA2b and RyR2), rather than the skeletal muscle isoforms (SERCA1 and RyR1), were found to be up-regulated. Based on these gene expression changes, the authors showed that inhibition of SERCA by thapsigargin decreased the β-adrenergic-induced increase in respiration [114]. Intriguingly, forced expression of PRDM16 in pig adipocytes, which lack functional UCP1, increased expression of a subset of genes associated with beige adipocytes. Moreover, down-regulation of SERCA2b in these cells reduced basal and β-adrenergic-induced respiration. These findings suggest that a futile Ca^2+^ cycling mechanism can operate in beige adipocytes, at least in the absence of UCP1.

Whilst extensive efforts have been made to explore the potential for exploiting BAT in treating obesity, to date direct evidence supporting this as a viable approach in humans is lacking. One problem is that activation of thermogenic adipocytes is thought to rely on β-adrenergic signalling, and so would be inherently non-specific. This lack of specificity includes serious negative consequences such as hypertension and increased risk of cardiovascular disease [243]. To identify alternative approaches to inducing thermogenic adipocyte response, Kajimura and colleagues set out to investigate the origin of beige adipocytes in mice lacking β-adrenergic signalling [133]. Consistent with previous findings [244,245]), either pharmacological blockage of β-AR signalling by propranolol, or genetic ablation in β-AR KO mice [246] had little effect on the adaptive thermogenic response to mild cold exposure [133,145,152]. Transcriptomic analysis showed that genes involved in skeletal muscle development, as well as those associated with beiging, were enriched in WAT isolated from β-AR KO mice compared with wild-type mice. Isolated stromal-vascular fraction (SVF) from mice treated with β-blocker contained a subset of cells expressing myogenic differentiation protein 1 (MYOD1) [133]. These cells, capable of forming MHC^+^ myotubes in culture, were later shown, using *MYOD1*-Cre^ERT2^ GFP-reporter mice, to form UCP1^+^ beige adipocytes *in vivo,* termed MYOD1^+^-derived beige fat [133]. Further analysis of beige adipocytes isolated from the MYOD1^+^ lineage led to the adoption of the name ‘glycolytic beige’ (g-beige), with significant enrichment of genes involved in glycolysis, glucose and carbohydrate metabolism distinct from both the classical beige and brown adipose signatures [133]. The proliferation of the smooth muscle actin (SMA)^+^; paired box gene 3 (PAX3)^+^; PDGFRα^+^; CD34^+^; CD29^+^ progenitor cell was restricted to iWAT, reflective perhaps of the increased heterogeneity and plasticity of this depot, and contributed substantially to whole organism glucose homeostasis. Ablation of MYOD1^+^-progenitors with diphtheria toxin substantially reduced g-beige formation, leading to reduced glucose uptake and oxygen consumption in WAT and impaired adaptive thermogenesis in response to cold exposure. This study also identified GA-binding protein α (GABPα) as a potent promotor of the differentiation of both MYOD1^+^ progenitors and C2C12 myoblasts (a mouse skeletal muscle cell line) to an adipocyte lineage. Moreover, GABPα was shown to be required for g-beige formation *in vivo* [133]. This implies that cold stress can recruit different progenitors, or induce a different differentiation pathway, depending on the level of β-adrenergic signalling. The beneficial effect of g-beige on glucose homeostasis has significant therapeutic potential, but it will be essential to first determine whether g-beige cells are present in humans.

Work from our group identified another type of beige-like adipocyte that we dubbed Skeletal-Muscle like AMP-activated protein kinase (AMPK) Reprogrammed Thermogenic (SMART) cells [134]. Widespread tissue expression of a gain-of-function AMPK mutant in mice led to the induction of SMART cells within the iWAT depot and this was associated with protection against high-fat diet-induced obesity through increased thermogenesis. Importantly, protection against diet-induced obesity was maintained when the mice were housed under thermoneutral conditions (for mice this is ∼30°C), implying that the effect was not reliant on UCP1-dependent thermogenesis. The SMART cells contain small, multilocular lipid droplets and are rich in mitochondria, similar to brown adipocytes. However, SMART cells do not express UCP1, distinguishing them from brown, canonical beige and glycolytic beige adipocytes. In response to a high-fat diet, there was a striking change in gene expression profiles between iWAT isolated from the AMPK gain-of-function mice compared with control mice. Expression of genes associated with striated muscle contraction, including SERCA1a, RyR1 and RyR3, was significantly increased in the gain-of-function mice. These results share obvious parallels with the findings from an earlier study that identified an increase in components of the Ca^2+^ cycling machinery in WAT of mice expressing PRDM16 in the absence of UCP1 [114]. It is worth noting that the two studies differed in the nature of the isoforms of SERCA and RyR that were up-regulated, with the cardiac isoforms increased in the PRDM16/UCP1 model and the skeletal muscle isoforms increased in the AMPK gain-of-function model.

An important finding in the AMPK gain-of-function model is the apparent bypass of UCP1 as a thermogenic pathway in WAT. Instead, thermogenesis is supported by increased mitochondrial ATP synthesis driving futile Ca^2+^ cycling mediated by SERCA1/RyR [134]. A previous study reported that pharmacological activation of AMPK promotes beiging in iWAT, with a concomitant increase in UCP1 protein expression, and a modest protection against high-fat diet-induced obesity [247]. In contrast with the genetic gain-of-function model, no evidence was presented to indicate that pharmacological activation of AMPK induced the expression of SMART cells. One possibility for the divergent phenotypes between the two studies could be differences in the degree and/or site of AMPK activation. Relevant to this point, selective expression of the gain-of-function AMPK mutant in mature adipocytes (using adiponectin-Cre) or classical white adipocyte progenitors (using PDGFRα-Cre) did not recapitulate the phenotype seen in the mice crossed with β-actin-Cre (to achieve widespread tissue expression) [134]. These findings suggest that induction of SMART cells requires AMPK activation in a specific, as yet unidentified, progenitor population. Further studies will be needed to identify these progenitor cells and to determine whether pharmacological activation of AMPK in these cells mimics the effect of genetic activation.

The gene signature of SMART cells includes increased expression of three of the four known myogenic regulatory factors (MYF5, MYF6 (also known as MRF4) and myogenin) suggesting that these cells also undergo a myogenic transition. This bears similarity with the g-beige cells, although it seems likely that the SMART cells have a lineage that is distinct from g-beige. This could also account for the difference in isoform expression of SERCA and RyR between SMART cells and UCP1 KO beige adipocytes as described by Ikeda et al. Finally, it is possible that AMPK activation drives the formation of bona fide brown adipocytes, rather than a ‘myogenic beige’, with suppression of UCP1 an independent action leading to the expression of compensatory thermogenic pathways.

As discussed above, several independent studies have identified novel adipocyte subtypes, with diverse functions and all of potential therapeutic benefit. The heterogeneity of adipose tissue, particularly with respect to lineage, is now the subject of intense scrutiny, as it would appear that recruitment of these cells is orchestrated primarily by pre-programmed responses. In vitro studies of these cells in culture provides a valuable approach to characterising their properties, but it will also be important to determine the contribution of the microenvironment in which they reside on their function. Many immune cells are known to modulate adipocyte function, and these processes are often disrupted in pathophysiological conditions [47,178,248,249]. Several reactive stromal populations have been identified which may contribute to adipocyte differentiation both during development and in cancer, providing a key link between tumour development and obesity [49,50]. To evaluate all aspects of adipocyte biology, new technologies, including refined lineage tracing and single-cell RNA sequencing, are being exploited to better characterise precursors and to identify fluctuations potentially linked to disease state [6,34,158,163,168,169,250,251].

## Understanding adipocyte lineage *in vivo*: new technology and future perspectives

**Figure 3.**
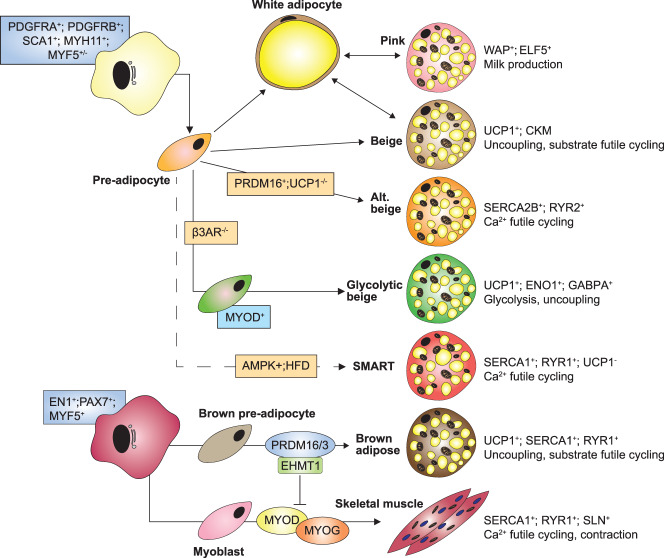
The heterogeneity and plasticity of adipocyte lineages. Key metabolic and thermogenic pathways operating in each cell type are shown together with predominant proteins involved in these pathways. Refer to the text for further details.

Extensive studies using lineage tracing have revealed the complexity and heterogeneity of pathways leading to the generation of adipocytes. The data generated from these studies, though often conflicting, have created a map of adipocyte lineage that is far more intricate than originally appreciated (Figure 3). Several reviews have consolidated these studies, with reference to the model used, the expression patterns observed and the inference of hierarchy within the stem cell niche [6,34,35,158,163,213]. However, the functional significance of lineage remains a key unanswered question. Given that functional differences exist between adipocytes of the same lineage, most notably beige and white, and even between neighbouring cells within a depot [34,158,163], it is unclear as to whether the origin of an adipocyte truly defines its function *in vivo*. Initial observations suggested that beige adipocytes were derived from transdifferentiation of pre-existing white adipocytes [252,253]. However, other studies indicated that most beige adipocytes stem from differentiation of precursor cells, rather than through transdifferentiation [129,254]. One example of where adipocyte transdifferentiation appears to play an important role is during lactation. The adipose tissue in mammary glands of female mice undergoes significant remodelling, with the generation of milk-producing alveolar cells containing mitochondria and large cytoplasmic lipid droplets, formed without proliferation of a progenitor, but instead derived from pre-existing white adipocytes [197,198].

The acquisition of a beige phenotype however is less defined, with evidence for transdifferentiation limited to the absence of proliferative events or the retention of a lineage-specific reporter in a morphologically distinct cell. Since many thermogenic adipocytes retain their lineage, as is the case between beige and white, assessment by common lineage markers such as PDGFRα/β does not distinguish a newly recruited cell from a pre-existing one. This is also true of white adipocytes that trace to a MYF5^+^ lineage, with some also retaining their primitive PAX3^+^ status in a mosaic-like fashion within one adipose depot [34]. At present no clear functional distinction between MYF5^+^ white adipocytes and classical PDGFRα^+^ adipocytes has been observed in the unchallenged state, with thermogenic gene expression similar to MYF5^-^ cells. Though no differential response to prolonged β3-AR stimulation was observed in these depots, deletion of PTEN led to a significant expansion of MYF5^+^ cells (BAT, retroperitoneal and interscapular WAT), with speculation that increased PI3K signalling, hyper insulin sensitivity and lipid accumulation conferred a metabolic advantage [35,255]. It has been shown that both transdifferentiation and *de novo* differentiation from precursor cells occurs in response to high fat diet and cold stress. This was demonstrated using a ‘MuralChaser’ lineage tracing system in which zinc finger protein 423 (ZFP423)^+^/PDGFRβ^+^ perivascular mural cells [181,182,256,257] were labelled with doxycycline-inducible ZFP423-GFP [181]. *De novo* adipocyte differentiation from ZFP423-GFP labelled mural cells was observed only after prolonged cold exposure, with the initial browning of the tissue independent of mural cell recruitment. These findings suggest the initial transformation from white to beige adipocytes involves either transdifferentiation of existing white adipocytes, or the recruitment of ZFP423 negative precursor cells. This multi-step process may explain the previously observed ‘harlequin’ patterning [34,211]. In this case, new cells are interspersed with existing cells from different lineages. Understanding the relevance of cell-type specific function and metabolism in these adipocyte lineages could help improve drug specificity and reduce off-target and potentially hazardous side effects, such as those incurred with β3 agonists.

**Figure 4.**
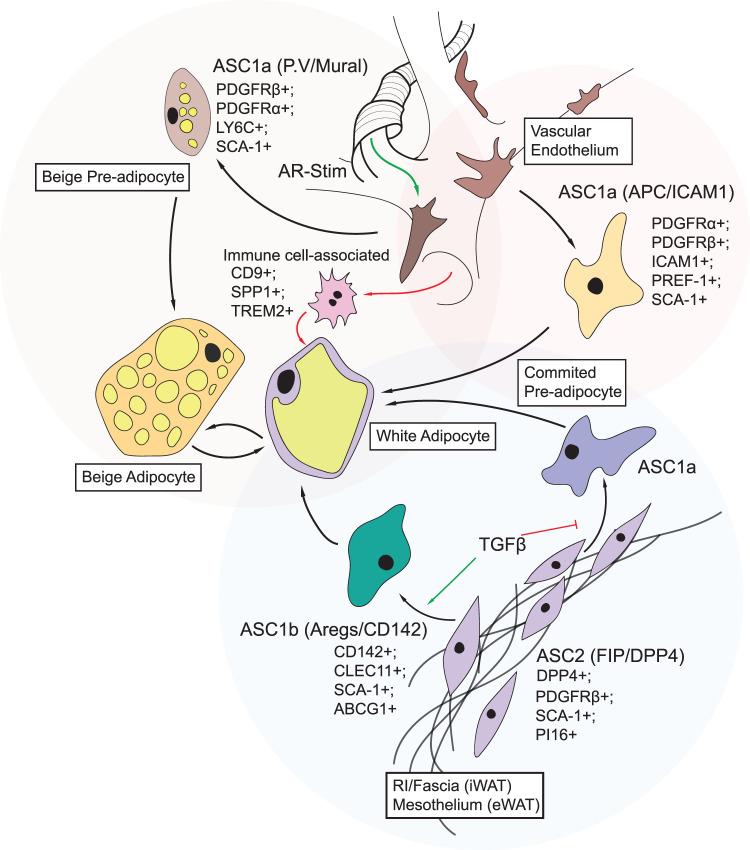
Contribution of known stem cell niches to mature adipocyte development. Adipose-stem cell (ASC) populations identified by single-cell RNA sequencing are shown with respect to proposed nomenclature and existing hierarchies [168,251,258,261–265]. ASC1, known formerly as Adipose Progenitor Cells (APCs) and committed pre-adipocytes, have been identified in all single-cell RNA sequencing studies reported, and give rise to mature adipocytes *in vivo.* They are further classified as ASC1a and ASC1b, with respect to their progenitor population. ASC1a, also known as APC and ICAM1/PREF-1 expressing pre-adipocytes are prevalent in most differentiated tissue, irrespective of depot. They encompass both PDGFRβ^+^ mural cells and PDGFRα^+^/PDGFRβ^−^ precursors, commonly associated with classical adipogenesis and are SCA1^+^. ASC1b, previously identified as CD142^+^/AREG adipocyte precursors are a distinct population, arising from a second master progenitor, ASC2. ASC2/DPP4^+^/FIP^+^ cells are of stromal origin, residing in the reticular interstitium (RI) of iWAT and mesothelium of eWAT). ASC2 cells give rise to both ASC1a and 1b populations, with TGFβ a potent lineage determinant between these cell fates. Immune cell populations contribute to the differentiation of ASC populations, with CD9^+^ macrophages expressing SPP1 and TREM2 found in crown-like structures surrounding mature adipocytes [251,265]. Functional differences identified between these populations suggest that all are adipogenic, with stimulus-specific recruitment under inflammatory and adrenergic stimuli.

More recently, single-cell RNA sequencing was used to identify distinct cell types in the SVF of both mouse and human adipose tissue [168,251,258–260]. A subset of these cells was found to reside in a new anatomically distinct structure within WAT, termed the reticular interstitium. Present within the reticular interstitium are the stromal cell precursors that are capable of differentiating into white adipocytes *in vivo* [168,258]. These findings challenge the idea that adipocyte precursors reside solely in the vasculature and peri-vascular regions. Instead, it is possible that adipocyte differentiation can stem from both a stromal (interstitial) mesenchymal dipeptidy peptidase 4 (DPP4)^+^/Wnt family member 2 (WNT2)^+^ progenitor [168], and from a PDGFRβ^+^ cell of peri-vascular origin [162,163,261]. The intermediary cells described by Merrick et al. [168] include the preadipocyte factor-1 (PREF-1)-expressing intercellular adhesion molecule 1 (ICAM1)^+^ pre-adipocytes, and the alternative CD142^+^/C-type lectin domain containing 11 (CLEC11)^+^ cells promoted by transforming growth factor β (TGFβ)–inhibition of ICAM1^+^ cells. Subsequently, additional populations of adipocytes were identified in humans with several clusters positively correlated with high mitochondrial content, oxidative metabolism and inversely correlated with disease state [258]. Though the attribution of function to these newly established hierarchies is not yet established, several inferences can be made, based on pre-existing understanding of adipose derived stem cell proliferation *in vivo*. Some of these links are shown in Figure 4 and have been reviewed recently [251].

## Future perspectives

In this article we have explored the interconversion of white and brown adipocytes and the basic functional consequences of adipocyte lineage. From the first identification of brown and beige adipose, researchers have been fascinated by the heterogeneity and plasticity of this abundant source of stem cells, with many applications beyond the treatment of metabolic disease. Easily accessible and with a lower rejection rate, adipose derived stem cells have been investigated for the treatment of ischemia [266] and stroke [267], to repair cartilage [268–270] and to generate stem cells for spinal injury and neurodegenerative disorder transplant therapy, through the production of neuron- and glial-like cells [271–273]. Whilst our understanding of adipocyte function has advanced significantly, we are only just beginning to explore the links between developmental origins and plasticity with respect to therapeutic potential. Future studies will undoubtedly build upon the large data-sets generated by -omics and single-cell techniques using targeted reporter systems, better-informed cell culture systems and refined imaging strategies to unpick the complex and diverse mechanisms governing adipose development. Based on these studies, we expect to see exciting new therapeutic interventions emerge based not just on small molecules, but perhaps on adipocyte stem cell therapy, for the treatment of metabolic disease.

## Open Access

Open access for this article was enabled by the participation of Imperial College London in an all-inclusive *Read & Publish* pilot with Portland Press and the Biochemical Society under a transformative agreement with JISC.

## Abbreviations

AMPK: AMP-activated protein kinase
AR: adrenergic receptor
BAT: brown adipose tissue
ER: endoplasmic reticulum
FFA: free fatty acid
FDG: fluoro-2-deoxy glucose
iWAT: inguinal white adipose tissue
KO: knockout
MAM: mitochondria-associated endoplasmic reticulum membrane
MYOD1: myogenic differentiation protein 1
MYF: myogenic regulatory factor
NST: non-shivering thermogenesis
PET: positron emission tomography
PDGFR: platelet-derived growth factor receptor
PRDM16: PR-domain containing 16
RyR: ryanodine receptor
scWAT: subcutaneous white adipose tissue
SERCA: sarco/endoplasmic reticulum Ca^2+^ ATPase
SLN: sarcolipin
SMART: skeletal muscle-like AMP-activated protein kinase reprogrammed thermogenic
SR: sarcoplasmic reticulum
SVF: stromal-vascular fraction
UCP1: uncoupling protein 1
WAT: white adipose tissue
ZFP423: zinc finger protein 423
